# Mechanical Characterization of Individual Needles in Microneedle Arrays: Factors Affecting Compression Test Results

**DOI:** 10.3390/pharmaceutics16111480

**Published:** 2024-11-20

**Authors:** Yusuke Tsuboko, Hideyuki Sakoda, Yoshihiro Okamoto, Yusuke Nomura, Eiichi Yamamoto

**Affiliations:** Division of Medical Devices, National Institute of Health Sciences, 3-25-26 Tonomachi, Kawasaki-ku, Kawasaki 210-9501, Kanagawa, Japan; tsuboko@nihs.go.jp (Y.T.); okamoto@nihs.go.jp (Y.O.); nomura@nihs.go.jp (Y.N.); eyamamoto@nihs.go.jp (E.Y.)

**Keywords:** buckling load, mechanical properties, single-needle compression, test condition, quality control

## Abstract

**Background:** This study aims to investigate the impact of test conditions on the results of the compression testing of microneedle arrays (MNAs). **Methods:** Uniaxial compression tests were conducted on polyglycolic acid-fabricated biodegradable MNAs. Load–displacement curves were obtained for varying conditions, including the number of microneedles (MNs) compressed simultaneously, compression speeds, and compression angles. Subsequently, the buckling load and stiffness were calculated, and the MN deformation during compression was observed. **Results:** The buckling load and stiffness per MN decreased significantly with a simultaneous increase in compressed MNs. The mean buckling load and stiffness of 52 MNs in single-needle compression tests were 0.211 ± 0.008 N and 13.9 ± 1.3 N/mm, respectively, with no variation among the three MNAs. However, a significant difference in buckling load and stiffness was observed among the MNs within the MNAs. Additionally, buckling loads and stiffnesses were significantly lower in certain MNs at the same location in different MNAs. Buckling load and stiffness decreased significantly during inclined compression compared to during vertical compression. While the tests evaluate the mechanical properties of MNAs, test results may vary depending on test conditions. **Conclusions:** Compression testing of the individual MNs comprising an MNA helps evaluate the mechanical properties of MNs and ensure the quality of MNAs.

## 1. Introduction

The development of microneedle arrays (MNAs) has progressed, and various forms of MNAs have been proposed. MNAs are now being actively developed for medical applications, including biosensing, fluid extraction, and transdermal drug delivery [1,2,3,4]. In regard to practical applications, MNA materials, concept and shape designs, and corresponding performance parameters, such as penetrability [5,6,7,8,9,10], material strength [11,12,13,14,15,16], solubility and drug elution [17], sterility [18,19,20], and stability [21,22,23], are evaluated. Notably, the strength of the microneedles (MNs) comprising an MNA is essential to ensure proper insertion into the skin and the intended delivery of the drug into the body. Therefore, compression testing is used to evaluate the mechanical strength of MNAs [6,24,25,26,27]. In addition to mechanical experiments, computational methods such as finite element analysis have been employed for geometric design and to predict puncture performance and mechanical strength [16,24,25,26,27,28,29].

Mechanical tests of MNAs should define their properties and ensure their safety, efficacy, and quality. Donnelly et al. [14] measured the MN compression force, the base-plate break strength and flexibility, and the transverse failure force of MN arrays. The regulatory review process will also require some evaluations of this type. Therefore, these tests must be reliable and reproducible. In most MNA compression tests, the buckling load and stiffness per MN are calculated from the peak load value and slope of the load–displacement curve divided by the number of simultaneously compressed MNs [14,15,27]. However, this method cannot evaluate the mechanical properties of the individual MNs that comprise the MNA. The potential issue is that the simultaneous compression of the entire MNA or some MNs may not be able to detect variations in the quality of each MN during manufacturing. Furthermore, few reports have investigated the effects of various test conditions on the results of mechanical tests [27].

In this study, we proposed a compression test to characterize the mechanical properties of individual MNs and investigate the influence of test conditions on the compression test results.

## 2. Materials and Methods

### 2.1. Test Samples

**Figure 1:**
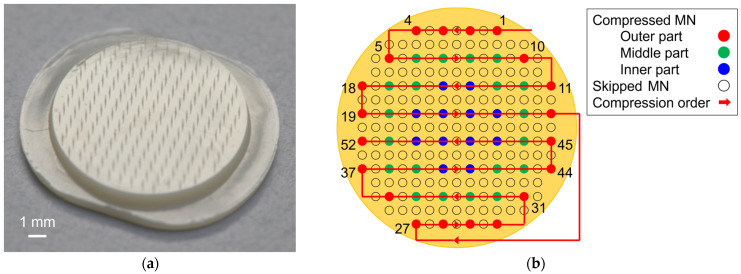
Microneedle arrays (MNAs) used in this study. (**a**) Appearance of the MNA consisting of 193 MNs; (**b**) Definition of microneedle (MN) numbers indicating their positions and classification by circumferential position.

An MNA consisting of 193 tapered MNs with a tip diameter of 40 µm, root diameter of 100 µm, and length of 600 µm was used (courtesy of CosMED Pharmaceutical Co., Ltd., Kyoto, Japan) (Figure 1a). The MNA was formed by melting biodegradable polyglycolic acid (PGA) with heat and then injecting it into a mold (injection molding). From each MNA, 52 MNs were tested (Figure 1b).

### 2.2. Experimental Setup

A universal testing machine (MST-X HS, Shimadzu Co., Kyoto, Japan) equipped with a 10 N load cell was employed for the compression tests. Two goniometric stages (TS-C411 and TS-C412, Chuo Precision Industrial Co., Ltd., Tokyo, Japan) were positioned orthogonally on the XY linear stage to adjust the compression angle, and the specimens were secured with double-sided adhesive tape. Stainless steel punches of various diameters were used as the compression jigs, depending on the test. In each test, the MN was aligned by operating the XY linear stage under the microscope so that the MN was in the center of the punch.

### 2.3. Compression Tests

A single-needle compression test was performed using a 0.5 mm diameter punch to investigate the characteristics of each MN that comprised the MNA. To simplify the test, among the 193 MNs on one MNA, 52 (every other MN from alternating lines) were compressed (Figure 1b). To investigate the effect of the number of MNs compressed simultaneously, in addition to a single MN (0.5 mm diameter punch), tests were also conducted with 4 MNs (1.4 mm), 9 MNs (2.1 mm), and 36 MNs (4.3 mm). Due to the limited number of specimens, 52, 16, 11, and 8 compression tests were performed for each condition. To evaluate how a needle’s base portion contributes to the MNA’s stiffness, compression tests of the base portion were performed using a 0.1 mm diameter punch, which was identical to the MNs’ root diameter. The standard compression speed was 0.5 mm/min. Compression tests were also conducted at speeds of 0.1, 2.0, and 10 mm/min, covering the entire practical range, to examine the effect of compression speed. Subsequently, MNs were compressed vertically (0°/0°) at 9.5°/0° and 9.5°/7.2° to assess the impact of compression angle. These angles were chosen as the maximum tilt angle of each of the two goniometric stages used in order to evaluate the worst-case scenario in the mechanical test. A digital optical microscope (VHX-5000, Keyence Corporation, Osaka, Japan) was used to position the specimens prior to testing and video-record the test in progress.

The load–displacement relationship obtained from the testing machine was recorded as time-series data. The maximum load value for each test was identified as the buckling load. Stiffness was calculated as the slope of the initial linear region of the load–displacement curve (between loads of 0.02 and 0.06 N). Subsequently, statistical analyses were performed on the acquired buckling load and stiffness data.

### 2.4. Statistical Analysis

One-way ANOVA, Tukey, and Tukey–Kramer tests were performed using Microsoft Excel 2021 and R version 4.3.1 for statistical analysis, and statistical significance was accepted if *p* < 0.05. Cohen’s d was calculated as the effect size for each test. When d < 0.2, 0.2 < d < 0.5, 0.5 < d < 0.8, and 0.8 < d, the effect size was considered very small, small, medium, and large, respectively.

## 3. Results

### 3.1. Effect of the Number of Needles Compressed Simultaneously

**Figure 2:**
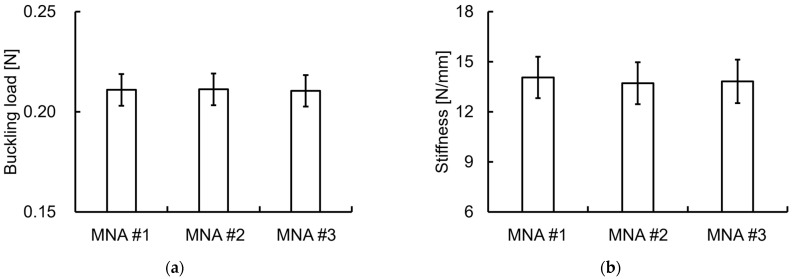
Comparison of mean buckling loads and stiffnesses among the three MNAs. (**a**) Mean buckling load; (**b**) stiffness. Results are expressed as mean ± SD.

Single-needle compression tests were performed on three MNAs from the same production lot (each with 52 MNs selected for testing), and the average buckling load and stiffness were calculated from the load–displacement curves. Buckling occurred near the tips of all the MNs. No differences in mean buckling load (0.211 ± 0.008 N) and stiffness (13.9 ± 1.3 N/mm) were found among the three MNAs of the same batch (Figure 2).

**Figure 3:**
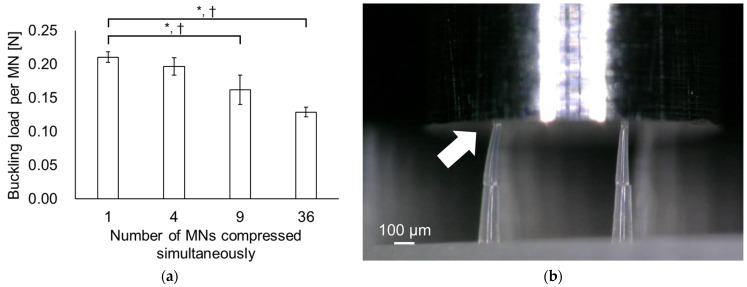
Effect of the number of MNs compressed simultaneously. (**a**) Buckling load per MN. Statistical comparisons were made using the Tukey-Kramer test; (**b**) microscopic observation of MNs buckling during compression testing of four MNs. White arrow indicates the MN that initiated buckling due to punch contact before other MNs. Results are expressed as mean ± SD. An asterisk indicates a significant difference at *p* < 0.05, and a dagger indicates that Cohen’s d > 0.8.

For the compression testing of 1, 4, 9, and 36 MNs, the buckling loads were 0.211 ± 0.008, 0.197 ± 0.052, 0.162 ± 0.022, and 0.129 ± 0.007 N, respectively (Figure 3a). As the number of simultaneously compressed MNs increased, the calculated buckling load per MN decreased. Video of multiple needle compressions revealed that the timing in which the punch made contact with the MNs was different for each MN, as was the timing of buckling when the maximum load occurred (Figure 3b).

### 3.2. Variation in Mechanical Properties Among MNAs and per MN for Single-Needle Compression

**Figure 4:**
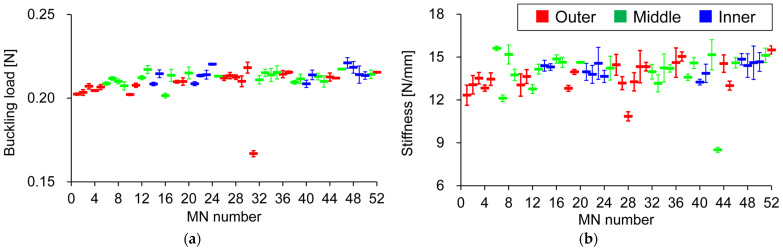
Position-dependent variation in mechanical properties of MNs. Mean (**a**) buckling load; (**b**) stiffness of individual MNs on three MNAs; MN numbers and circumferential MN distributions plotted in blue (inner), green (middle), and red (outer) correspond to Figure 1b. Statistical comparisons were made using one-way analysis of variance (ANOVA). Results are expressed as mean ± SD.

**Figure 5:**
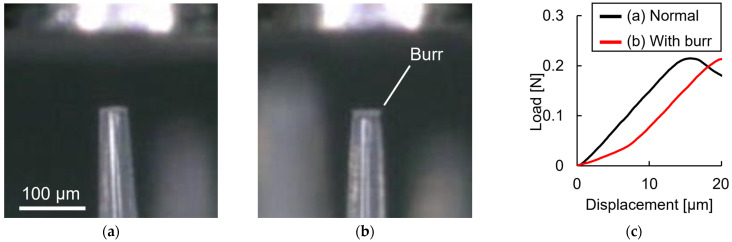
Relationship between MNs’ tip conditions and load–displacement curves. (**a**) Normal MN (#27); (**b**) MN with burrs (#43); (**c**) comparison of load–displacement curve in MNs with and without burring.

The Shapiro–Wilk test was conducted to verify that each MN’s buckling load and rigidity in the three MNAs follow a normal distribution. Normality was confirmed at *p* < 0.05. The 52 MN distribution pattern of MNAs was classified into inner, middle, and outer parts, and these were compared; however, the circumferential position had no obvious effect. Instead, as found by the one-way ANOVA, the MNs varied significantly (Figure 4). In addition, the results of Tukey’s multiple comparison indicate that certain MNs clearly had low buckling loads (MN #31: 0.167 ± 0.001 N) and stiffnesses (#28: 14.3 ± 0.2 N/mm, #43: 8.51 ± 0.15 N/mm). Burrs less than 10 µm in height were observed at the tips of MNs with significantly low stiffnesses (Figure 5).

### 3.3. Effect of Base Deformation

**Figure 6:**
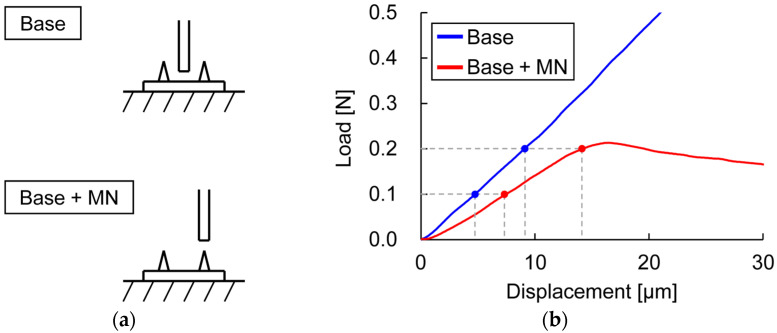
Effect of base portion on load–displacement curves. (**a**) Schemas for compression of base only, and entire MN including base; (**b**) load–displacement curve in each condition.

The compressions of the entire MN, including the base portion, and the base portion only were compared (Figure 6). For compressive loads of 0.1 and 0.2 N (immediately before buckling), the displacement ratios in tests of the base only and the entire MN were 64.0 and 63.9%, respectively, and >50% of the deformation was due to base deformation (Table 1).

### 3.4. Effects of Compression Speed and Angle

**Figure 7:**
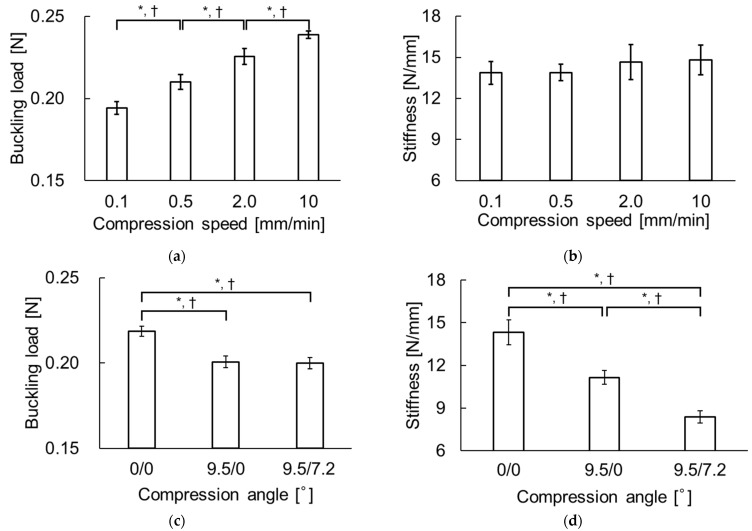
Effects of compression speed on (**a**) buckling load and (**b**) stiffness; effects of compression angle on (**c**) buckling load and (**d**) stiffness. Statistical comparisons were made using the Tukey test. Results are expressed as mean ± SD. An asterisk indicates a significant difference at *p* < 0.05, and a dagger indicates that Cohen’s d > 0.8.

The buckling load significantly increased (0.194 ± 0.004, 0.210 ± 0.004, 0.226 ± 0.005, and 0.239 ± 0.002 N) as compression speed increased (0.1, 0.5, 2.0, and 10 mm/min) (Figure 7a), while stiffness did not significantly differ (Figure 7b). The buckling loads and stiffnesses in the inclined compression groups (9.5°/0° and 9.5°/7.2°) were significantly lower than those in the vertical (0°/0°) group (Figure 7c,d). For compression angles of 0°/0°, 9.5°/0°, and 9.5°/7.2°, the buckling loads were 0.219 ± 0.003, 0.201 ± 0.003, and 0.200 ± 0.003 N, and the stiffnesses were 14.3 ± 0.9, 11.2 ± 0.5, and 8.40 ± 0.43 N/mm, respectively.

## 4. Discussion

This study investigated the influence of test conditions on the results of compression tests aimed at characterizing MNAs.

**Figure 8:**
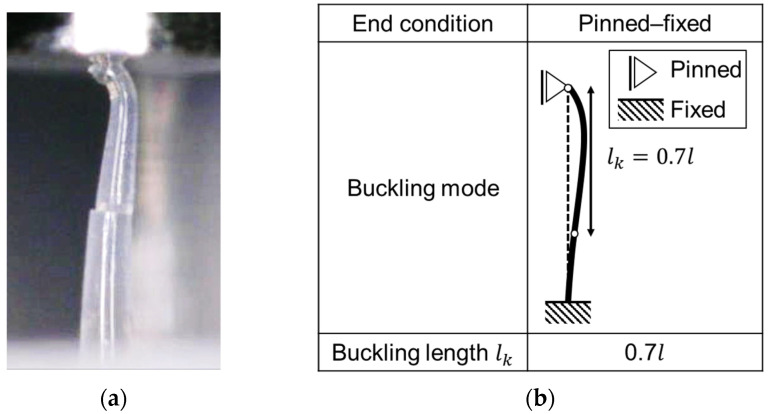
Buckling of MN in single-needle compression and buckling mode. (**a**) Buckling near the tip of the MN (#28); (**b**) scheme of buckling mode in the pinned–fixed condition.

Microscopic observation of the MNAs during the test can help identify the failure mode and the source of variation in the test results. When compressive loads were applied to the MNAs, the MNs were in the pinned–fixed buckling mode (Figure 8) and did not slip on the punch surface. This finding is consistent with that of Park and Prausnitz [11]. Needle tip slippage is a potential source of variation in the test results. The mechanical properties of the MNs were evaluated regarding stiffness and strength. The load increased almost linearly until buckling, at which point the maximum load was reached, and none of the MNs broke. Notably, the stiffness values obtained include the effect of the deformation of the base portion in addition to that of the MN; therefore, the results of the compression tests of the MNAs are also affected by changes in the material properties and design of the base portion (Figure 6).

Owing to discrepancies in the buckling timing of each MN, the calculated buckling load per MN significantly decreased as the number of simultaneously compressed MNs increased. The same tendency has also been reported when dissolving MNAs [27]. From the load–displacement curve, it was inferred that a difference of a few micrometers in the height of individual MNs is sufficient for timing discrepancies to occur. In MNA manufacturing, slight height discrepancies that can be caused by fabrication errors, burrs, and slightly distorted base portions are considered unavoidable. Expressed differently, the compression test results of multiple MNs may not properly evaluate the mechanical properties of the MNs because, as described above, they are affected by factors other than the mechanical properties of the MNs. 

Our proposed single-needle compression test avoids the above problems and can properly characterize the mechanical properties of MNs. In addition, it can detect and evaluate the variations in individual MNs that cannot be detected when compression testing multiple MNs. The experimental sample was confirmed to show no differences in the mechanical properties among the MNAs, but significant differences were found among their MNs. In addition, the MNs in certain positions exhibited characteristic strength and stiffness values that were reproduced in different MNAs, suggesting very high manufacturing reproducibility for the MNA products used in this study. This means that the variation between MNs within an MNA may be greater than the variation among MNAs, indicating the importance of testing individual MNs. However, since only one product lot was tested in this study, the reproducibility across different lots must still be verified. Compression testing of individual MNs revealed that burrs at the MN tip could also be detected based on the calculated stiffness values. Even when MNs had burrs, the latter slope of the load–displacement curve did not change, so it was assumed that there was no difference in the intrinsic stiffness of the MN. However, the detection of burrs may be necessary because their presence may affect the penetrability of the MNA.

The speed of compression has been shown to affect the strength results. This is due to the material’s viscoelasticity, and therefore, speed’s influence varies from material to material. Consequently, it is difficult to compare tests performed at different test speeds. Conversely, the compression speed had no clear effect on stiffness, perhaps because its effect was not significantly greater than the variation in the MNs’ quality or the stiffness at the base. The variation in the mechanical properties of MNs at specific locations in different MNAs was less than the variation between different MNs within the same MNA, so identifying individual MNs may allow for more sensitive comparative testing.

The angle at which the specimens were placed affected the test results, especially the stiffness. Although the test results were obtained under fairly extreme conditions, care should be taken in terms of alignment when placing the specimens in the testing machine, and mechanical evaluation should consider the possibility that vertical MNA punctures may not occur in actual applications.

This study examined the effect of test conditions on the results of mechanical tests on one particular product. Ando et al. [27] reported the effects of differences in compression between single and multiple needles in dissolving MNAs. Haider et al. [30] also conducted transverse load tests on each MN individually. The importance of single-needle compression testing as a method of evaluating MNAs for the purpose of optimizing MNA manufacturing methods, manufacturing equipment, and processes, including dissolving and hollow types, is expected to increase. The presence or absence and the degree of influence on the results of the various factors and conditions of mechanical tests may vary depending on the material and design of the product being evaluated. For example, Gittard et al. [6] and Park and Prausnitz [11] reported that whether an MN exhibits the maximum load at buckling depends on its aspect ratio. Therefore, when evaluating the mechanical properties of MNAs, it is desirable to consider the influences of various factors and conditions on the results of mechanical tests.

## 5. Conclusions

This study confirms that the results of compression tests to evaluate the mechanical properties of MNAs are affected by factors such as the number of MNs tested simultaneously, compression speed, compression angle, and the deformation of the base portion. These factors should be considered when performing mechanical tests to ensure a high level of reliability and reproducibility. The results of the tests performed under different test conditions should be compared carefully. In addition, the mechanical properties of MNAs cannot be properly evaluated in tests that compress multiple MNs simultaneously. Evaluation of the individual MNs that comprise the MNA provides valuable insight into the effects of variations in needle quality during manufacturing, as well as the mechanical properties of the MNs. Implementation of single-needle compression tests within quality control ensures the consistent quality of MNA products.

## Figures and Tables

**Table 1:** Base deformation: effect of compression.

Compressive Load [N]	Displacement [µm]		Displacement Ratio (Base/Base + MN) [%]
	Base	Base + MN	
0.1	4.75	7.42	64.0
0.2	9.08	14.2	63.9

## Data Availability

Data is contained within the article.
